# Interventions to reduce inequalities in vaccine uptake in children and adolescents aged <19 years: a systematic review

**DOI:** 10.1136/jech-2016-207572

**Published:** 2016-08-17

**Authors:** Tim Crocker-Buque, Michael Edelstein, Sandra Mounier-Jack

**Affiliations:** 1 Health Protection Research Unit in Immunisation, Faculty of Public Health and Policy, London School of Hygiene and Tropical Medicine, London, UK; 2 Department of Immunisation, Hepatitis and Blood Safety, Public Health England, London, UK

**Keywords:** VACCINATION, IMMUNIZATION, INEQUALITIES, PRIMARY CARE, COMMUNICABLE DISEASES

## Abstract

**Background:**

In high-income countries, substantial differences exist in vaccine uptake relating to socioeconomic status, gender, ethnic group, geographic location and religious belief. This paper updates a 2009 systematic review on effective interventions to decrease vaccine uptake inequalities in light of new technologies applied to vaccination and new vaccine programmes (eg, human papillomavirus in adolescents).

**Methods:**

We searched MEDLINE, Embase, ASSIA, The Campbell Collaboration, CINAHL, The Cochrane Database of Systematic Reviews, Eppi Centre, Eric and PsychINFO for intervention, cohort or ecological studies conducted at primary/community care level in children and young people from birth to 19 years in OECD countries, with vaccine uptake or coverage as outcomes, published between 2008 and 2015.

**Results:**

The 41 included studies evaluated complex multicomponent interventions (n=16), reminder/recall systems (n=18), outreach programmes (n=3) or computer-based interventions (n=2). Complex, locally designed interventions demonstrated the best evidence for effectiveness in reducing inequalities in deprived, urban, ethnically diverse communities. There is some evidence that postal and telephone reminders are effective, however, evidence remains mixed for text-message reminders, although these may be more effective in adolescents. Interventions that escalated in intensity appeared particularly effective. Computer-based interventions were not effective. Few studies targeted an inequality specifically, although several reported differential effects by the ethnic group.

**Conclusions:**

Locally designed, multicomponent interventions should be used in urban, ethnically diverse, deprived populations. Some evidence is emerging for text-message reminders, particularly in adolescents. Further research should be conducted in the UK and Europe with a focus on reducing specific inequalities.

## Introduction

In high-income countries, substantial differences exist in vaccine uptake relating to socioeconomic status, gender, ethnic group, geographic location and religious belief.^1–24^ In 2009, the National Institute for Health and Care Excellence (NICE) conducted a systematic review of effectiveness and cost-effectiveness of interventions to ‘reduce differences in the uptake of immunisations in children and young people under the age of 19 years’.^19^ Since then new technologies have emerged, including data systems and online interventions, and have been applied to vaccination. In addition, new programmes have been implemented, such as human papillomavirus (HPV) vaccine in adolescents. An updated review of the evidence is therefore warranted. The aim of this study is to update the 2009 NICE systematic review, focusing and refining the recommendations on effective interventions to decrease vaccine uptake inequalities in high-income countries.

## Materials and Methods

We repeated the NICE guidance methodology,^19^ ^25^ conducting our review in line with the Preferred Reporting Items for Systematic Reviews and Meta-Analysis (PRISMA) statement.^26^

### Search strategy

We searched MEDLINE, Embase, ASSIA, The Campbell Collaboration, CINAHL, The Cochrane Database of Systematic Reviews, Eppi Centre, Eric and PsychINFO using the strategy described in online supplementary appendix 1. Results were limited to publications in English from April 2008 until November 2015.

10.1136/jech-2016-207572.supp1 supplementary appendix

### Inclusion and exclusion criteria

We included studies with the following characteristics:

- *Study design*: randomised controlled trials (RCTs), quasi-experimental (including interrupted time series and before-and-after studies), ecological and observational cohort studies.
- *Population*: children and young people (CYP) from birth to 19 years in OECD countries.^27^
- *Intervention*: delivered at primary/community care level, with the aim of increasing vaccine uptake in a specific population or in the overall population, with outcomes reported for specific subgroups.
- *Outcomes*: vaccine uptake, including initiation of vaccination course, schedule completion, being up-to-date (UTD) for age, or coverage, with either a focus on reducing inequalities or where outcomes in different population groups are reported.

In addition, we included references from review articles or protocols identified in the search that fitted inclusion criteria, but we did not consider inequalities.

### Study selection process

One reviewer initially screened articles on title and manually de-duplicated records. Two reviewers screened potentially relevant abstracts independently. Any disagreement was resolved by discussion on the basis of the inclusion criteria. Both reviewers agreed the final inclusions.

## Results

The study selection process is presented in online supplementary figure S1. Of 12 386 unique articles, 315 abstracts were screened. Of these, 80 full text articles were reviewed, along with 23 studies identified from the references of 22 review articles or protocols.^28–49^

10.1136/jech-2016-207572.supp2 supplementary figure PRISMA flowchart of literature selection

In total, 41 studies were included (17 RCTs, 20 quasi-experimental and 4 retrospective cohort studies), which were conducted in the USA (n=31), the UK (n=5), Canada (n=3) and Australia (n=2).

Studies reported on multicomponent complex interventions (n=16), patient-focused reminder/recall systems (n=18), outreach programmes (n=3), prompts for healthcare workers (HCWs) (n=2) and computer-based interventions (n=2).

We categorised results by using the intervention type and by vaccinations for the age group:

- Childhood vaccines from birth to age 11 (singly or in combination): tetanus, diphtheria and pertussis (TDaP); polio (IPV); haemophilus influenza b (Hib); pneumococcal (PCV); rotavirus; meningitis B (MenB); meningitis C (MenC); measles, mumps and rubella (MMR).
- Adolescent vaccines from age 11 to 19 (singly or in combination): HPV, MenC, quadrivalent meningitis (Men4) and relevant boosters.
- Seasonal influenza alone in CYP.

The terms ‘uptake’ and ‘coverage’ were used inconsistently in the literature. We have defined ‘uptake’ as the proportion of the eligible population who received a vaccine during a specific time period and ‘coverage’ as the proportion of an eligible population that is vaccinated, regardless of when they received the vaccine.

### Complex interventions

Complex interventions comprise several interacting components that may impact on a range of outcomes or have variability in delivery.^50^ Table 1 describes intervention components, sample size and study outcomes.

**Table 1:** Population, intervention components and outcomes of the included studies considering complex interventions

References	First author and year	Population	Vaccine (s)	Inequality	Sample (intervention)	Intervention name	Intervention components										Outcome (effect measures and/or 95% CI)
							Identification or targeting of CYP in risk groups	Promotional materials (eg, posters or media campaign)	Education children's parents or young people directly	Patient reminder/recall and/or tracking and surveillance	Outreach (eg, home visits)	HCW training (. allied professionals)	HCW prompts	Additional services (eg, clinics)	Standing Orders*	Community involvement	
51	Findley *et al*, 2008	Children 19–35 months	Schedule	Urban, ethnicity, low income	10 857 (895)	Start right	–	Y	Y	Y	Y	Y	–	–	–	Y	11.1% higher uptake and 53% more likely to be UTD (p<0.01, no CI)
52	Fu *et al*, 2012	Children aged <24 months	Schedule	Urban, ethnicity, low income	3945 (1999)	–	–	Y	Y	Y	–	Y	Y	Y	Y	–	16% increase in uptake to 87% (p<0.001, no CI)
53	Suryadevara *et al*, 2013	CYP<19 years	Schedule	Low income/deprived	1531	–	Y	–	Y	Y	–	–	–	Y	–	Y	Increase in coverage In enrolled children from 28% to 40%
54	Isaac *et al*, 2015	High-risk infants identified at birth	Schedule	Urban, ethnicity, low income	9746 (4562)	Family First	Y	–	Y	–	Y	–	–	–	–	–	Intervention risk ratio 1.06 for being UTD (CI 1.03 to 1.08).
55	Hambidge *et al*, 2009	New-born infants until 15 months old	Schedule	Urban, ethnicity, low income	811	–	Y	Y	Y	Y	Y	–	–	–	–	–	Intervention OR of 1.6 for being UTD (CI 1.2 to 2.1)
56	Cockman *et al*, 2011	Children aged 2 years	MMR	Urban, ethnicity, low income	36 practices	–	Y	–	–	Y	–	Y	Y	Y	–	–	A significant quarterly coverage increase of 1.86%
57	Thomas *et al*, 2008	Aboriginal infants	7-valent PCV	Ethnicity	Ecological	–	Y	Y	Y	–	Y	Y	Y	–	–	–	10% increase in coverage to 50% (no statistical analysis)
58, 59	Potts *et al*., 2014; Sinka *et al*, 2013.	Girls aged 12–13, with catch-up for under 18s.	HPV	Deprivation	220 000	–	Y	Y	–	Y	–	Y	–	Y	–	–	Equal uptake by deprivation quintile for first dose, with uptake reducing for doses 2 and 3.
60	Cates *et al*, 2011	Girls aged 9–19	HPV	Urban, low income, ethnicity	100 counties (4 counties)	–	–	Y	–	–	–	–	Y	–	–	Y	Different responses across intervention sites, no significant difference overall.
61	Cates *et al*, 2014	Men aged 11–12	HPV	Urban, low income, ethnicity	28 counties (13 counties)	Protect Him	–	Y	–	–	–	Y	–	–	–	–	Intervention 34% more likely to be vaccinated (p<0.002), with higher uptake in non-Hispanic black population.
62	Moss *et al*, 2012	Adolescents aged 12–17	Schedule	Urban, low income, ethnicity	17 health centres	AFIX (by proxy)	–	–	–	–	–	Y (to increase use of complex intervention)	–	–	–	–	Intervention 1.1% increase in being UTD across the schedule 1 month later (to 32.2%, p=0.001)
63	Chung *et al*, 2015	Adolescents aged 11–18	Tdap, Men, HPV	Urban/rural	5 counties (1 county, 7 health centres)	–	–	Y	–	Y	–	Y	–	–	–	–	Increase in the first dose HPV in 11–12 year olds (OR 1.21, CI 1.01 to 1.50), quad men (OR 2.23, CI 1.7 to 2.9) and in 13 to 19 year olds HPV vaccine completion in men (OR 1.45, CI 1.02 to 2.05).
64, 65	Nowalk *et al*, 2014; Zimmerman *et al*, 2014	CYP aged 6 months to 18 years	Influenza	Ethnicity	24 practices	Four Pillars	–	Y	–	Y	Y	Y	Y	Y	Y	–	Greater increase in uptake in intervention group (9.9% vs 4.2% in controls, p<0.001) and higher in white children (16.7%, p<0.05)
66	Logue *et al* 2011	Children and adults aged >6 months	Influenza	Urban, low income, ethnicity	5061	–	–	Y	–	–	–	Y	Y	–	Y	–	Increase in uptake in 3–8 year olds (15%, p<0.05) and 9–17 year olds (19%, p<0.05), but not younger children.

*Standing orders allow non-prescribing health professionals to give medicines including vaccinations without a doctor's prescription in certain situations.

CYP, children and young people; HCW, healthcare workers, for example, doctors, nurses or allied health professionals; HPV, human papillomavirus vaccination; MMR, measles, mumps and rubella vaccination; PCV, pneumococcal vaccination; UTD, up-to-date with all recommended vaccines for age.

#### Childhood vaccinations

Six studies showed evidence of effectiveness for locally developed complex interventions to increase uptake in ethnically diverse, low-income populations. In the USA, a retrospective evaluation of ‘Start Right’, a community-developed intervention involving bilingual promotional materials, peer health educators, outreach, parental reminders and provider support, found that children aged 19–35 months enrolled in the programme had significantly higher uptake than control children.^51^ Another intervention involving reminder/recall systems, increased clinic access, use of standing orders and provision of educational materials was evaluated in a before-and-after study, which found that children in the intervention year had a statistically significant increase in vaccine uptake.^52^ An intervention identifying children not UTD attending a charitable community organisation for resource-poor families, providing information and vaccinations, followed by reminders, increased coverage rates after 9 months.^53^ In Canada, an evaluation of the ‘Families First’ programme (involving identification of high-risk families, home visiting and signposting to health services) found small but significant increases in being UTD by first and second birthdays.^54^ An RCT evaluating an intervention that escalated in intensity on the basis of vaccine status over time, which involved universal, language appropriate reminder postcards, targeted telephone calls and intensive outreach and home visitation, showed a significant increase in children being UTD at 12 months.^55^ In the UK, a complex primary care focused intervention (developing a general practitioner (GP) network, financial incentives, better use of data and IT) significantly increased uptake of MMR coverage in a deprived, diverse community, although inequalities persisted in some smaller ethnic groups.^56^

The uncontrolled evaluation of an intervention to increase PCV coverage in Aboriginal infants in an Australian urban community (involving staff training, information materials, contact with parents, see online supplementary information, and stickers in health records) showed an increase.^57^ However, no statistical analysis was performed and coverage remained under the national Aboriginal average.

#### Adolescent vaccinations

Two Scottish studies reported on the HPV vaccine programme national roll-out among females aged 12 and 13, alongside a time-limited catch-up programme for females aged up to 18 (in school and in the community) and an accompanying media campaign.^58^ ^59^ In the routine and catch-up programmes, uptake decreased by deprivation quintile for each subsequent dose, leading to a greater proportion of more deprived young people not completing the programme and thus increasing inequalities. Uptake was lowest in females who had left school and were vaccinated in the community (dose 1: 49%, dose 3: 30%), who were also more likely to be in a lower socioeconomic group. First-dose uptake was higher when regional health boards delivered the community catch-up clinic (52.3%), compared to GP practices (43.5%).

Two US studies used social marketing to increase HPV vaccine uptake in a large geographic area with an urban–rural divide, high ethnic diversity and a large low-income population: one in females aged 9–19 years and one in males aged 9–13 years.^60^ ^61^ In females, the approach overall had no differential effect. However, males who were unvaccinated in intervention counties were significantly more likely to be vaccinated after 6 months, with higher uptake among the non-Hispanic black population. However, males in intervention counties were also 24% less likely to receive a TDaP booster (p≤0.001).

A large before-and-after American study involving 17 federally qualified health centres (that act as a safety-net healthcare provider for underserved communities) evaluated the impact of a webinar targeting clinic coordinators, aiming to increase implementation of AFIX, a CDC-recommended list of practice-based interventions to increase vaccine uptake (including data collection and analysis, feedback to providers, incentive and specific staff). It found a statistically significant 1.1% increase in adolescents becoming UTD.^62^

Another American study evaluated a practice-based intervention (involving educational meetings, reminder/recall system usage, targeted reminders and incentive payments) alongside a telephone reminder to parents delivered through schools, which sought to reduce an urban/rural inequality.^63^ Results showed a significant increase in the uptake of first-dose HPV and Men4 vaccines (11–12 year olds) and HPV vaccine course completion (males aged 13–18 years). No significant differences found for other age bands or vaccine types.

#### Influenza vaccine in CYP

Two related American articles reported on the ‘Four Pillars’ intervention (increased service access, reminder/recall system, improved provider office systems and immunisation champions) to increase the uptake of seasonal influenza vaccine in CYP aged 6 months to 18 years.^64^ ^65^ Increases were seen in intervention and control groups, with significantly greater increases in intervention areas and in white children, with a narrowing of the gap between ethnic groups.

One further uncontrolled before-and-after study in an urban, ethnically diverse family medicine centre examined a predominantly HCW-focused intervention (involving policy change, standing orders, health record modification and information to patients) and showed increases in coverage in children aged between 3 and 18 (but not younger), with greater increases in African-Americans.^66^

### Outreach programmes

One study conducted in parents of children from birth to 35 months evaluated the ‘BIRTH PIP’ intervention (parental education at birth followed by home visits) among 400 African-American mothers.^67^ When compared with the population, participants had significantly higher vaccination coverage (92% vs 49%), although there was significant loss to follow-up (50% loss by 19 months). Another RCT evaluating an enhanced prenatal and postnatal home visitation programme among 530 low-income women versus regular community care found no difference in vaccination uptake.^68^ However, one other US study found that children living in deprived areas with an immunisation coordinator were less likely not to be UTD for age (adjusted OR 0.6) and that overall disparities had decreased between groups over the time of the programme.^69^

### Reminder/recall systems

Table 2 describes the intervention type, sample size and study outcomes.

**Table 2:** Population, intervention components and outcomes of the included studies considering reminder/recall interventions targeted at patients or clients.

References	First author and year	Population	Vaccine(s)	Inequality	Sample Size (intervention)	Intervention components					Intervention description	Outcome (effect measures and/or 95% CI)
						Identification of those not UTD*	Text message(s)	Letter(s) to home	Telephone call(s)	Outreach (eg, home visit)		
70	Kempe *et al*, 2013	Children aged 19–35 months	Schedule	Urban/rural, not UTD	55 173	Y	–	Y	–	–	Practices participating received financial assistance. Up to three notifications sent.	Increase in chances of becoming UTD if population based reminder system used (relative risk 1.23, CI 1.10 to 1.37)
71	Kempe *et al*, 2015	Children aged 19–35 months	Schedule	Urban/rural, not UTD	18 235	Y		Y	Y	–	Centralised reminder system involved either telephone and letters or letters alone. The practice-based system was variable at practice level, but involved calls or letters or both.	Increase in children being UTD by 2.5% (p<0.001) using the centralised system (adj OR 1.31, CI 1.16 to 1.48)
72	Atchison *et al*, 2013	Children under 5 years	Schedule	Urban, low income, ethnicity	32 practices	Y	–	Y	Y	Y	Escalating intervention comprising two letters, followed by a telephone call or home visit if no response.	Significant increase in proportion UTD in the intervention group, but as a result of unexplained decreases in the non-intervention group.
73	Dombkowski *et al*, 2014	Children under 20 months	Schedule	Urban, not UTD	10 175	Y	–	Y	–	–	Recall notices issued at 7 and 19 months, with a reminder notice at 12 months.	No difference in children at 7 or 12 months, but a significant difference of 7% (p<0.0001)| at 19 months.
74	Lemstra *et al*, 2011	Children not UTD with MMR at 24 months	MMR	Deprivation, low income	629	Y	–	–	Y	Y	Home visits targeted as a separate intervention in low-income areas.	Significant increase in intervention areas (rate ratio 1.10, CI 1.08 to 1.12). Increase in home visit areas, but not significant due to small numbers.
75	Cushon *et al*, 2012	Children aged 14–20 months	MMR	Deprivation, low-income	24 540	Y	–	Y	Y	Y	Identification of children not UTD, five telephone calls, letter home and then home visitation.	Increases observed in across all study sites, including low-income areas. No significant difference observed in intervention sites, disparities remained.
76	Stockwell *et al*, 2012 A	Children aged 7–22 months	Hib	Urban, low income	174	Y	Y	Y	–	–	Repeated reminders delivered five times until vaccination status registered as UTD.	Non-significant difference, possibly due to small sample size (n=174)
77	Hofstetter *et al*, 2015 A	Children aged 9.5–10.5 months.	MMR	Urban, low income, ethnicity	2054	–	Y	–	–	–	Participants either received reminders to schedule a vaccination appointment and then an appointment reminder; appointment reminder only; or usual care.	No difference between arms except in children with no vaccination appointment booked, who received scheduling and appointment reminders (relative risk ratio 1.11, CI 1.00 to 1.24)
78	Abbott *et al*, 2013	Aboriginal children from birth to 20 months	Schedule	Ethnicity	505	–	–	–	–	–	Reminder calendar given to parents	Significant increase in vaccinations being given on time, once outliers were excluded.
76	Stockwell *et al*, 2012 A	Adolescents aged 11–18	Td, Men4	Urban, low income, ethnicity	361 (195)	Y	Y	–	–	–	Repeated reminders delivered five times until vaccination status registered as UTD.	Significantly more adolescents in the intervention arm received missing vaccines at 4, 12 and 24 weeks (eg, at 12 weeks 26.7% vs 13.9% in controls, 12.8% difference CI 4.7% to 20.9%, p=0.003).
79	Kharbanda *et al*, 2011	Adolescent girls aged 9–20	HPV (doses 2 and 3)	Urban	124	Y	Y	–	–	–	Up to three weekly reminders that child due for an HPV dose.	Intervention individuals were more likely than controls, contemporaneous (adjusted OR 2.03, CI 1.29 to 3.22 p=0.003) and historical (AOR 1.83, CI 1.23 to 2.71, p=0.002) to receive next HPV dose on time.
80	Szilagyi *et al*, 2011	Adolescents aged 11–15	Pertussis, Men, HPV	Ethnicity	7546	Y	–	Y	Y	Y	Reminder/recall and home visits undertaken by specialist vaccine system navigators.	Becoming UTD for each vaccine was 12% to 16% higher in the intervention group (p<0.001), with 71% of the intervention group having received a reminder and 12% a home visit.
81	Bar-Shain *et al*, 2015	Adolescents aged 11–18	HPV, MenC, Tdap	Deprivation, ethnicity	3393	Y	Y	Y	Y	–	Depending on availability of contact information either an email, text message or postcard was sent, repeated every 2 months for up to 12 months until UTD	25.5% of adolescents in the study received at least one missing vaccine and response to the messaging reduced with each round. There were no differential effects by age, gender, insurance status or ethnicity.
82	Brigham *et al*, 2012	Adolescents aged 13–17.	Tdap, Men4	Urban, not UTD	424	Y	–	–	Y	–	Compared calls to parents to calls to parents and adolescents.	Higher uptake in the parent and adolescent reminder group (adj OR 2.27,) however with a large CI (CI 1.00 to 5.18)
83	Morris *et al*, 2015	Adolescents aged 11–17.	HPV, Men4, Tdap, Var	Urban, deprivation	5050	Y	Y	Y	Y	–	Series of 3 batches of reminders over 6 months, based on parents’ choice of message medium.	Those who signed up for any method of reminder were more likely to become UTD than those who only received an enrolment phone call (24.6% vs 12.4%, p<0.001).
84	Mantzari *et al*, 2015	Adolescent girls aged 17–18	HPV initiation and completion	Deprivation	1000	Y	Y	Y	–	–	Letter with incentive offer sent to house, followed by series of text messages between the second and third dose.	Increased uptake of the first dose in intervention individuals (OR 1.63). However, no differential impact by deprivation.
85	Stockwell *et al*, 2012 B	CYP aged 6 months to 18 years.	Influenza	Low income, ethnicity	9213	–	Y				Series of five text messages with educational information.	Higher proportion of CYP vaccinated in the intervention group (3.7% increase, CI 1.5% to 5.9%, p=0.001; relative risk ratio 1.09, CI 1.04 to 1.15), although overall rates remained low at around 40%
86	Stockwell *et al*, 2015	Children 6 months to 8 years	Influenza	Low income, ethnicity	660	–	Y	Y	–	–	Three arms: education vs conventional text plus letter, and usual care (letter only) control.	Children in the educational group were significantly more likely to receive the second influenza dose (72.7%, p=0.003) compared to conventional text (66.7%) and postal reminder only (57.1%).
87	Hofstetter *et al*, 2015 B	CYP 6 months to 17 years	Influenza	Low income, ethnicity	5462	Y	Y	Y	–	–	Three arms: interactive educational message vs educational text vs usual care control.	The interactive component of the messages had low uptake (1.0% using the service); however, slightly more in this arm were vaccinated than those who received the education only text (38.5% vs 35.3%, relative risk ratio 1.09 CI 1.00 to 1.19, p=0.04)

CYP, children and young people; HCW, healthcare workers, for example, doctors, nurses or allied health professionals; Hib, *Haemophilus influenzae* group b vaccination; HPV, human papillomavirus vaccination; Men4, quadrivalent meningococcal vaccination (A, C, W and Y); MenC, meningococcal group c vaccination; MMR, measles, mumps and rubella vaccination; Td, tetanus and diphtheria vaccination; Tdap, tetanus, diphtheria, pertussis vaccination; UTD, up-to-date with all recommended vaccines for age; Var, varicella vaccination.

#### Childhood vaccinations

Two large American RCTs compared centralised systems versus Practice-based reminder/recall systems and concluded that centralised systems increased likelihood of children becoming UTD for age.^70^ ^71^

A UK before-and-after study evaluating an escalating reminder/recall system, including letters and home visits, in an ethnically diverse, urban population, found that uptake was stable in intervention areas, but decreased in non-intervention areas.^72^ In the USA, a large RCT targeting non-UTD children aged <20 months with a postal reminder or recall notices found no coverage difference in younger children (7 or 12 months), but a significant increase at 19 months.^73^ The hypothesis offered was that younger children would be attending services more regularly and thus have higher uptake, whereas older children might not and thus be more responsive to reminders. Of two Canadian studies examining MMR coverage in deprived areas, one controlled before-and-after study found that telephone reminders increased MMR uptake in children not UTD at 24 months;^74^ however, the other non-controlled time series found increases in intervention (targeted phone, mail and outreach) and non-intervention sites, with no decrease in socioeconomic disparities.^75^

The Text4Health study evaluated the effect of sending text messages to parents in an American, urban, low-income population, prompting them to have their children aged 7–22 months vaccinated with Hib.^76^ It found a non-significant uptake difference after 2 weeks. An RCT undertaken in an urban, low-income minority ethnic population in the USA randomised participants to receive text-message reminders to schedule an appointment and/or reminders of the appointment details, or usual care to increase MMR vaccine uptake at 13 months.^77^ There was no difference in uptake between the arms, except in children who did not have a vaccination appointment booked and who received scheduling and appointment reminders.

An uncontrolled before-and-after study evaluating an immunisation reminder calendar given to parents of Aboriginal children in Australia showed timeliness for being UTD for vaccines increased, once significant outliers were excluded.^78^

#### Adolescent vaccinations

Two studies examined the use of repeated SMS reminders. The Text4Health study found significant increases in MenC and TDaP vaccine uptake among 11–18 year olds in the intervention arm.^76^ Another non-randomised trial looked at second and third doses of HPV vaccine in urban adolescent females and found that intervention individuals were significantly more likely to receive doses on time.^79^

Two studies examined different reminder/recall media. One RCT evaluated a tiered protocol with progressively more intensive reminder/recall and outreach dependent on continued lack of vaccine uptake.^80^ It found that the intervention was associated with becoming UTD for each vaccine and was more successful among females and black and Hispanic adolescents. An uncontrolled study targeting ethnically and socioeconomically diverse parents of adolescents not UTD with a variety of reminders over 12 months showed that 25.5% participants received one missing vaccine.^81^

An RCT comparing uptake of Men4 and TDaP in adolescents not UTD using phone reminders only to parents, versus parents and adolescents, found significantly higher uptake in the parent and adolescent reminder group.^82^ A non-RCT compared postal, email or SMS reminders for adolescent vaccination on the basis of parental preference and found that those who signed up were more likely to become UTD, irrespective of the method of reminder.^83^

A UK study evaluated giving a £40 incentive alongside a reminder/recall system and found significantly increased odds of completing the HPV vaccine course, irrespective of deprivation levels.^84^

#### Influenza vaccination in CYP

Three American RCTs examined the effect of SMS reminders targeted at low-income, minority ethnic parents on influenza vaccine uptake. Parents of CYP aged 6 months to 18 years receiving 5 weekly community-developed educational and clinic reminder text messages significantly increased uptake although overall levels remained low.^85^ When comparing educational and conventional SMSs with postal reminders targeted to parents of children aged 6months to 8 years, those receiving the educational SMS had higher second-dose influenza vaccine uptake.^86^ Another study compared interactive SMSs with educational ones, compared to usual care, in CYP aged 6 months to 17 years unvaccinated for influenza late in the season.^87^ Children of parents who received an interactive SMS were slightly more likely to be vaccinated. However, only 1% parents used the interactive feature.

### Reminder systems targeted at HCWs

A large retrospective before-and-after study in the USA examined the effect of a vaccine alert placed within electronic health records of females aged 9–26.^88^ The intervention prompted cohort had higher initiation than the unprompted control cohort (35% vs 21.3%), with higher initiation rates seen in African-Americans. Another American RCT examined the effect of HCW prompts on adolescent vaccine uptake in a diverse population, but found no difference in uptake between intervention and control practices.^89^

### Computer-based interventions

Two studies examining computer-based interventions found no effect on vaccine uptake. An RCT evaluating an intervention targeting African-American females to increase HPV vaccine uptake (‘Girls on Guard’) found that only 12% of 216 participants initiated the vaccine course, with equal numbers in intervention and control groups.^90^ Another randomised study examined a computer-based health message intervention delivered in school-based clinics in a population of ethnically diverse parents of non-HPV vaccinated children (n=445) and found that rhetorical questioning message prompts increased vaccination intention, but not uptake.^91^

## Discussion

The impact of socioeconomic context, including deprivation, ethnicity or geography, on health outcomes has been well documented^92^ and is equally true of vaccine programmes.^93^ Presented here is the evidence of effectiveness for interventions to reduce the resulting inequalities in vaccination coverage. Multicomponent locally designed interventions demonstrated the best evidence in children and adolescents in the short term. These interventions are designed for a specific context and population, so may not be transferable to other settings. The 2009 NICE guidance recommended home visiting as a possibly cost-effective intervention, which is partially supported by this evidence. All nine interventions that included a home visit component showed some evidence of effectiveness.^51^ ^54^ ^55^ ^57^ ^64^ ^72^ ^74^ ^75^ ^81^ Although two of three studies considering outreach interventions alone were not effective, they were either small or had significant loss to follow-up.^67^ ^68^ The three studies using escalating intervention intensity seemed particularly effective, which is consistent with the previous review.^55^ ^72^ ^80^ This may be a cost-effective way of incorporating home visiting into a programme. Social marketing interventions show mixed evidence, but could be a promising approach in adolescents.^60^ ^61^ No studies provided good long-term evidence of sustained uptake.

The evidence around reminder/recall systems continues to evolve. In the USA, centralised reminder/recall systems worked better than practice-based ones; however, this may be specific to the American health system. Evidence of effectiveness of text-message reminders in reducing inequalities remains limited. The type of messages received may impact vaccine uptake, particularly if educational or interactive messages are used. However, more research is required to confirm this effect. A recent systematic review of ‘new media’ to improve vaccine uptake found evidence of effectiveness for SMS reminders, but also considered a wide variety of other interventions such as mobile phone apps and the use of social media.^44^ We did not identify any studies that used new media to reduce vaccine uptake inequalities, and this could form potentially useful future work. The two studies examining computer-based behaviour change interventions found no evidence of effectiveness.

There is some evidence for postal and telephone reminders in children and adolescents, although heterogeneity of interventions precludes from drawing firm conclusions. Choosing the reminder method and including adolescents alongside parents for reminders possibly improved effectiveness.^82^ ^83^ A recent systematic review found that targeting postal and telephone reminders to parents was most effective at increasing early childhood vaccination.^37^

We found mixed evidence for HCW-focused reminders, which adds to the previous review's two positive studies. The evidence for client-side financial incentives was mixed in the previous review, and we found one additional study that showed an increase in adolescent HPV uptake. However, a recent systematic review found no effect of incentives on vaccine uptake in children.^30^

Two studies noted intervention effectiveness in older children, but not younger children.^66^ ^73^ This may be because younger children are more likely to seek routine healthcare and should be a consideration when targeting interventions.

### Tackling inequalities

Most interventions did not specifically target inequalities, but instead delivered interventions in low-uptake populations and focused on CYP not UTD for age.

Several interventions reported differential effects by ethnicity, including Aboriginal infants in Australia,^57^ ^78^ White children^64–66^ and non-Hispanic black adolescents^65^ as well as black and Hispanic adolescents in the USA.^61^ ^80^ These interventions are very context and population specific, and further work is required to develop the evidence base for interventions targeting specific ethnic groups or other characteristics associated with vaccine uptake inequality such as deprivation.

### Limitations

Studies were mainly from the USA, with some from the UK, Canada and Australia. We found none from other European countries. This paucity mirrors the low number of European studies in the previous review. This may be related to the English language restriction or due to the unavailability of certain types of data (eg, it is illegal to collect data on ethnicity in France). We did not consider cost-effectiveness of interventions, although this was reported in some studies, due to challenges in comparing results between different health systems. Vaccine hesitancy was not considered for two reasons: first, a separate systematic review exists on interventions to reduce hesitancy and^40^ second, very few inclusions in that review or this paper measured uptake or coverage as an outcome. There are likely to be opportunities to incorporate evidence-based interventions to reduce hesitancy more explicitly within interventions to reduce inequalities in uptake between different groups.

### Recommendations

- Locally designed, multicomponent interventions have the strongest evidence for increasing vaccine uptake, particularly in urban, ethnically diverse, low-income or deprived population.
- Some evidence is emerging relating to the use of text messages and other types of reminder/recall systems, particularly in adolescents, and should be considered.
- Interventions that increase in intensity targeting persistent non-responders have some evidence of effectiveness and may be more cost-effective than other interventions, such as universal home visiting alone.
- Further research should be conducted: in the UK and Europe, focusing on reducing specific inequalities, such as by the ethnic or religious group and on smartphone technology to increase vaccine uptake.

**What is already known on this subject**

- In high-income countries, substantial differences exist in vaccine uptake relating to socioeconomic status, gender, ethnic group, geographic location and religious belief. A previous systematic review from 2009 concluded that the evidence was promising for outreach programmes, mixed for reminder/recall systems and information provision and limited for text messages and service delivery interventions.

**What this study adds**

- This study updates the systematic review to 2015 and concludes that locally designed, multicomponent interventions have evidence of effectiveness in urban, ethnically diverse, deprived populations. There is some evidence emerging for text-message reminders, particularly in adolescents, but that other types of technology have not yet been evaluated.
