# Supplementary material for: Interventions to reduce inequalities in vaccine uptake in children and adolescents aged <19 years: a systematic review
Source: J Epidemiol Community Health. 2016 Aug 17;71(1):87–97. doi: 10.1136/jech-2016-207572 (PMC5256276; doi:10.1136/jech-2016-207572)
Supplement: supplementary appendix [file jech-2016-207572supp_appendix.pdf]

APPENDIX 1 – Search Strategy

1. exp Child/ (2450193)
2. exp Infant/ (987875)
3. exp Adolescent/ (1331416)
4. (child$ or baby or babies or adolescent$ or teenage$ or youth or young person or young people or youngster or pupil$ or student$ or school$ or playground$ or playgroup$ or nurser$ or kindergarten$).mp. (3524025)
5. 1 or 2 or 3 or 4 (4004225)
6. ((improve$ or enhance$ or encourage$ or increase$ or support or assist$ or maximi$ or promot$) adj2 (uptake or coverage or cover or acceptanc$ or complianc$ or adoption or rate$ or access or equit$ or equalit$)).mp. (237777)
7. ((decreas$ or reduc$ or minimi$ or detect$ or change$) adj2 (inequalit$ or unequal or inequit$ or disparit$ or imparit$ or varia$ or unevenness or discrepanc$ or imbalance$ or differen$ or barrier$)).mp. (132793)
8. ((geographic or regional or urban or rural or ethnic$ or religiou$ or class or socioeconomic or social or economic or demographic or cultural) adj2 (inequalit$ or unequal or inequit$ or disparit$ or imparit$ or varia$ or unevenness or discrepanc$ or imbalance$ or differen$ or barrier$)).mp. (146286)
9.(poor or income or unemploy$ or middle class$ or working class$ or disadvantaged or socially excluded or inner city or poverty or deprived or vulnerable or jobless or immigra$ or asylum seek$ or refugee or single parent or single mother or lone parent$ or lone mother$ or disabled or disabilit$ or handicap$).mp. (1106665)
10. (social housing or council hous$ or council estate or temporary accommodation or foster home or orphanage or looked-after child$ or housing benefit or social services or social work$ or social security).mp. (57301)
11. (minorit$ or ethnic$ or black or white or asian$ or indian$ or pakistani$ or bangladeshi$ or african$ or caribbean or chinese or oriental or turk$ or arab or gyps$ or roman$ or traveller$ or religiou$ or christian$ or catholic$ or protestant$ or jew$ or muslim$ or hindu$ or sikh$ or jehova$).mp. (1313370)
12. exp Socioeconomic Factors/ (201999)
13. exp Unemployment/ (13133)
14. exp Urban Population/ (38544)
15. exp Suburban Population/ (341)
16. exp Rural Population/ (35098)
17. exp Vulnerable Populations/ (8996)
18. exp Single Parent/ (2539)
19. exp Disabled Children/ (8147)
20. exp social welfare/ or exp social work/ (38694)
21. exp Public Housing/ (19980)
22. exp Minority Groups/ (12267)
23. exp Religion/ (61038)
24. exp ethnic groups/ (94935)
25. exp continental population groups/ (195765)
26. 6 or 7 or 8 or 9 or 10 or 11 or 12 or 13 or 14 or 15 or 16 or 17 or 18 or 19 or 20 or 21 or 22 or 23 or 24 or 25 (2948182)
27. exp Vaccination/ (144953)
28. exp immunization programs/ (23476)
29. exp immunization/ (257400)
30. (immuni$ not innate).tw. (277361)
31. 27 or 28 or 29 or 30 (447010)
32. 5 and 26 and 31 (19249)
33. exp travel/ (32241)
34. 32 not 33 (18867)
35. limit 34 to (english language and yr="2008 -Current") (9171)
