# Supplementary figures and images for: Interventions to reduce inequalities in vaccine uptake in children and adolescents aged <19 years: a systematic review
Source: J Epidemiol Community Health. 2016 Aug 17;71(1):87–97. doi: 10.1136/jech-2016-207572 (PMC5256276; doi:10.1136/jech-2016-207572)

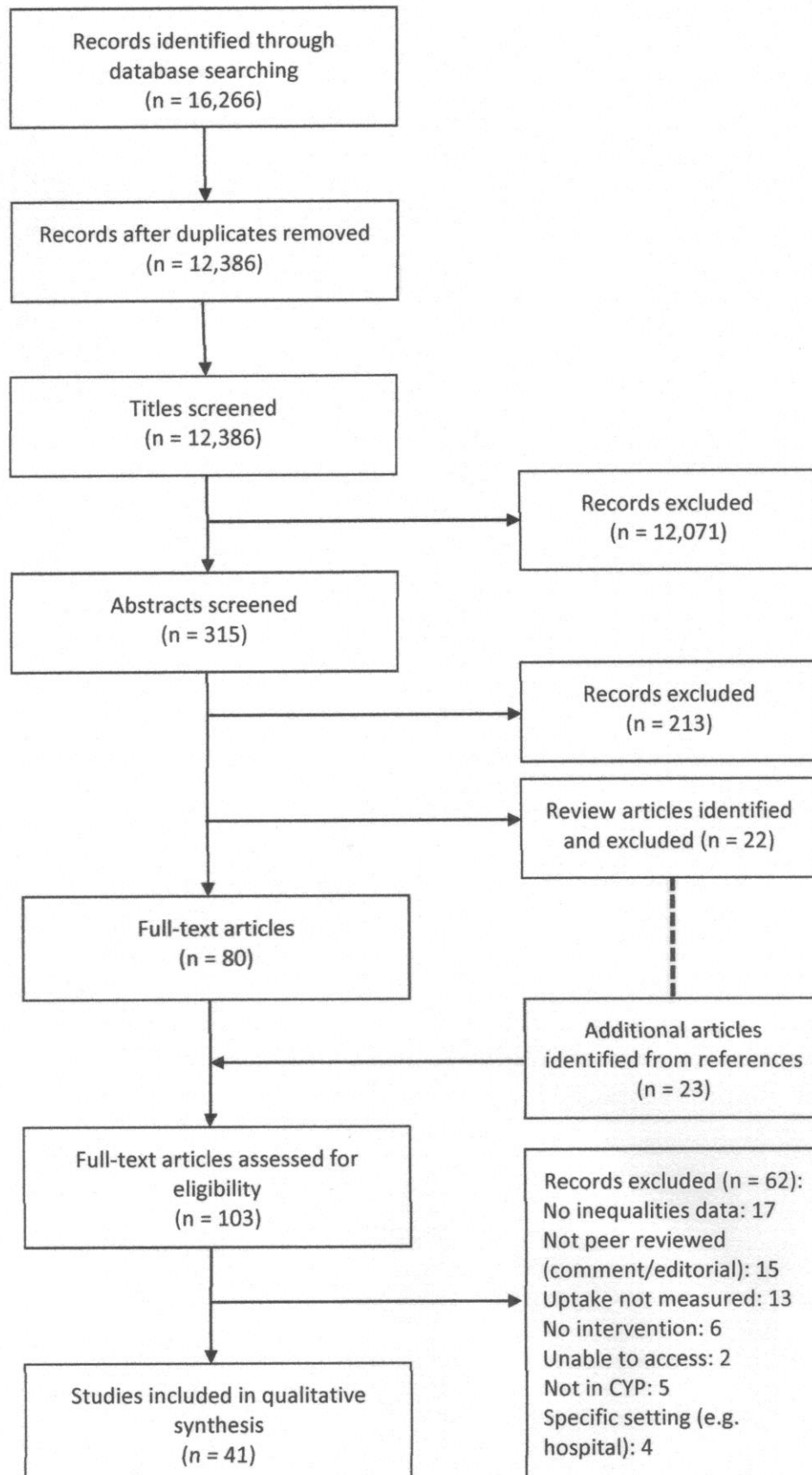

**Figure 1:** PRISMA flowchart of literature selection

Supplement: supplementary figure — PRISMA flowchart of literature selection [file jech-2016-207572supp_figure.pdf]
